# Peer-Assisted Learning in a Gross Anatomy Dissection Course

**DOI:** 10.1371/journal.pone.0142988

**Published:** 2015-11-13

**Authors:** Eui-Ryoung Han, Eun-Kyung Chung, Kwang-Il Nam

**Affiliations:** 1 Office of Education and Research, Chonnam National University Hospital, Gwangju, South Korea; 2 Department of Medical Education, Chonnam National University Medical School, Gwangju, South Korea; 3 Department of Anatomy, Chonnam National University Medical School, Gwangju, South Korea; NYIT College of Osteopathic Medicine, UNITED STATES

## Abstract

Peer-assisted learning encourages students to participate more actively in the dissection process and promotes thoughtful dissection. We implemented peer-assisted dissection in 2012 and compared its effects on students’ self-assessments of learning and their academic achievement with those of faculty-led dissection. All subjects performed dissections after a lecture about upper-limb gross anatomy. Experimental group (*n* = 134) dissected a cadaver while guided by peer tutors who had prepared for the dissection in advance, and control group (*n* = 71) dissected a cadaver after the introduction by a faculty via prosection. Self-assessment scores regarding the learning objectives related to upper limbs were significantly higher in experimental group than in control group. Additionally, experimental group received significantly higher academic scores than did control group. The students in peer-assisted learning perceived themselves as having a better understanding of course content and achieved better academic results compared with those who participated in faculty-led dissection. Peer-assisted dissection contributed to self-perception and to the ability to retain and explain anatomical knowledge.

## Introduction

Cadaver dissection is an irreplaceable part of anatomy education that enriches the student experience through a close encounter with human mortality [1–3]. Hands-on dissection is necessary for appreciating the complexity and multidimensionality of the human body, for demonstrating the breadth of anatomical variation, and for learning the basic language of medicine [1–4]. Cadaver dissection not only provides opportunities for hands-on learning, but also offers a forum for students to engage in content rich discussions and to assist in each other’s learning [5]. Nevertheless, dissection has become a lengthy and tedious activity in which much of the time is spent watching somebody else dissect [6]. In this respect, cadaver dissection is in need of a change in the teaching and learning method and peer-assisted learning may be an effective alternative.

As a form of cooperative learning, peer-assisted learning promotes more active engagement by students in the learning process and prevents them from leaving the course [7]. Peer tutoring enhances cognitive processing of the tutors by increasing attention to and motivation for learning [7]. Peer tutors acquire positive attitudes toward learning, become more confident in their ability to teach and communicate content, and experience the satisfaction of helping others [8]. The tutees also benefit from peer-assisted learning. The students who are taught by similar social groups perceive the freedom to express opinions and the ownership of the learning process [7, 9].

Several reports have compared the effect of peer-assisted versus faculty-led learning, and indicated that students’ academic achievement after peer-assisted learning was as effective as that of faculty-led learning in clinical skills training and problem-based curricula in health sciences or law [9–11]. However, there have been inconclusive results to show whether students in a peer-assisted dissection model perform better when considering objective measurements than those in faculty-led dissection [12–15]. We implemented peer-assisted learning to encourage the medical students’ participation and interactive learning in the dissection. We hypothesized that peer-assisted cadaver dissection would be as effective as faculty-led dissection in terms of students’ perceptions and retention of anatomical knowledge.

## Materials and Methods

### Study participants and dissection course

The study subjects consisted of students who were in their first year of medical school at Chonnam National University Medical School in 2012 and 2008 and who had been trained in gross anatomy dissection during their first semester. All subjects had completed a lecture about upper-limb gross anatomy as part of the standard curriculum. The dissection course consisted of three 2-h sessions designed to independently address the shoulder joint, axilla, elbow joint, forearm, wrist joint, and hand. An intact cadaver was allocated to every subgroup during the dissection course.

Experimental group consisted of 134 students who were in their first year of medical school in 2012 and who participated in peer-assisted learning in the dissection laboratory. This group was divided into 20 subgroups (average size: 6.7 students) and three subgroups took the roles of tutors in the other subgroups by rotation at every dissection course. In other words, twenty-one students in three subgroups served as tutors, and one or two tutors were assigned to each of the other 17 subgroups during the dissection course. The roles of students were then switched at the next course. Tutors dissected the intact parts of the upper limb in advance with the guidance of textbooks and video clips; they could ask questions to a faculty member if they were facing difficulties. Members of the three tutor subgroups provided a 10-minute explanation of this practice to each tutee subgroup before the start of the dissection. The explanation addressed issues such as important anatomical structures and how to perform a dissection and identified the structures that should be carefully manipulated. During the dissection course itself, one or two tutors assisted each of 17 subgroups while they performed the dissections.

Control group consisted of 71 students who were in their first year of medical school in 2008 and who were randomly assigned to faculty-led learning groups involving prosections. They were divided into 10 subgroups. A student representative from each subgroup reviewed the parts being dissected with the faculty member using a prosection. After a 30-min introduction covering topics that would subsequently be addressed in the course, each representative shared this information with other students in her/his subgroup during the dissection course. All students of each subgroup participated actively in the dissection of the entire cadavers.

The Chonnam National University Hospital Institutional Review Board determined that the study was exempt from human subject research regulations. Participation in the survey was voluntary, and students were informed that their answers were confidential. Written consent was obtained from all participants.

### Data collection

All participating students were asked to complete a short questionnaire requesting demographic information and to rate their knowledge with regard to the upper-limb learning objectives. The learning objectives were addressed by 12 items based on the core anatomy syllabus [16] and the learning objectives for medical students [17]. Students were asked to indicate the degree to which they agreed with each item using a five-point Likert scale (1 = strongly disagree, 5 = strongly agree).

Immediately after completing the lectures and dissection courses, all students completed a written examination that consisted of multiple-choice, short-answer, and essay questions. The identical faculty, who delivered the lectures and guided the dissection courses in two groups, set the exam questions based on the learning objectives. The examination in 2012 was conducted in the same way as that in 2008, to compare academic results between two groups. The subjects in 2012 were blinded to the fact that their academic results would be compared with those of the students in 2008.

### Data analysis

We performed a chi-square test to assess similarities and differences in the general characteristics of the participants. A Mann–Whitney *U*-test was used to compare self-perceptions regarding learning objectives and academic achievement between groups. All analyses were performed using SPSS software version 21.0 (SPSS, Inc., Chicago, IL, USA).

## Results

The general characteristics of all participants are presented in Table 1. The two groups did not differ significantly with regard to gender or age.

**Table 1:** General characteristics of study participants.

	Experimental group	Control group	χ^2^ or t	*p-value*
	(Peer-assisted dissection)	(Faculty-led dissection)		
Gender			0.654	*0*.*419*
Male	81(60.4)	47(66.2)		
Female	53(39.6)	24(33.8)		
Age	24.5±2.9	24.5±2.7	-0.104	*0*.*917*

unit: no.(%), mean±SD

The self-assessment scores on all but one item of the 12-item questionnaire addressing upper-limb learning objectives were significantly higher in experimental group, which performed peer-assisted dissection, than in control group, which performed faculty-led dissection. With the exception of the ability to describe the position and function of the retinacula of the wrist and the tendon sheaths of the wrist and hand, students in experimental group perceived themselves as having a better understanding of the major structures and their courses and functions than did those in control group (Table 2).

**Table 2:** Comparison of self-assessment scores regarding the upper-limb learning objectives between groups.

Learning objectives	Experimental group	Control group	*p-value* *
	(Peer-assisted dissection)	(Faculty-led dissection)	
Describe the fascial compartments delimiting the major muscle groups of the upper limbs.	3.63±0.70	3.21±0.74	*0*.*000*
Describe the origin, course and distribution of the major arteries and their branches	3.64±0.64	3.29±0.75	*0*.*002*
Describe the courses of the main veins of the upper limbs	3.75±0.74	3.33±0.70	*0*.*000*
Describe the organization of brachial plexus, its origin in the neck and continuation to the axilla and upper limb	3.99±0.74	3.08±0.92	*0*.*000*
Describe the origin, course and function of the axillary, radial, musculocutaneous, median and ulnar nerves in the arm, fore-arm, wrist and hand	3.51±0.73	3.10±0.84	*0*.*001*
Describe the boundaries of the axilla	4.10±0.83	3.17±0.93	*0*.*000*
Describe the movements of the pectoral girdle	3.16±0.86	2.77±0.97	*0*.*018*
Describe the factors that contribute to the stability of the shoulder joint	3.99±0.75	3.23±0.90	*0*.*000*
Describe the anatomy of the elbow joint	3.96±0.73	3.57±0.82	*0*.*001*
Describe the anatomy of the wrist	3.75±0.80	3.44±0.86	*0*.*022*
Name and demonstrate the movements of the fingers and thumb	3.69±0.91	3.25±0.72	*0*.*001*
Describe the position and function of the retinacula of the wrist and the tendon sheaths of the wrist and hand	3.61±0.81	3.48±0.82	*0*.*274*

five-point Likert scale; unit: mean±SD

* by Mann–Whitney *U*-test.

The total self-assessment scores were also higher in experimental group than in control group. Additionally, experimental group achieved significantly higher academic scores than did control group (Table 3).

**Table 3:** Comparison of self-assessment and academic achievement scores between groups.

	Experimental group	Control group	*p-value* *
	(Peer-assisted dissection)	(Faculty-led dissection)	
Total self-assessment score	44.79±4.81	38.97±5.32	*0*.*000*
Academic achievement score	73.75±16.87	69.03±16.76	*0*.*029*

* by Mann–Whitney *U*-test.

Maximum score: Total self-assessment score, 60; Academic achievement score, 100; unit: mean±SD

## Discussion

Peer-assisted learning in a dissection course enhanced medical students’ assessment in their understanding of the multidimensional structure of the human body and led to better academic results compared with faculty-led dissection. Specifically, peer tutoring experiences increase students’ understanding of course content and their retention of the information they taught to their peers [18].

The stereotype of anatomy as endless memorization can cause students to approach dissection with a negative attitude [19]. However, students with high levels of motivation and positive attitudes toward dissection tend to devote more time to and engage more actively in this task [20]. Peer-assisted learning is thought to enhance students’ attention to and motivation for the dissection and encourages them perceive their experience as more active and interesting [7]. Hendelman et al. [21] reported that most students believed that they learned more about, developed better attitudes toward, and acquired better skills including communication and self-study skills in anatomy via peer-assisted than via faculty-led learning. Additionally, we postulated that academic achievement would also be superior following peer-assisted than faculty-led learning.

This effect of peer-assisted learning was predictable insofar as this approach allows more time for the tutors to responsibly prepare, improve their teaching and communication skills, and to become more confident in self-directed learning [18]. In contrast, some tutees worried about the competence of peer tutors or their dependence on tutors. Other studies have found similar results regarding students’ complaints about peer-assisted learning [7, 14, 15, 21, 22]. However, even less-capable tutors functioned well in this role, doing their best and treating the tutees with empathy [8]. Equally, the tutees guided by students tutors tend to spend more time on self-study to compensate for their tutors lack of expertise [9, 15]. Students felt more relaxed during discussions with peer tutors than during those with faculty members, rating the immediate feedback they received from the peers more favorably than that received from the faculty [23, 24]. Eventually, the tutees viewed the dissection course as more interactive and as promoting greater ownership of the learning process [7]. The negative comments about peer-assisted learning seem to be caused by concerns about what might happen rather than by actual experiences [12]. Additionally, the negative comments may have resulted from the intentionally random assignment of students to the dissection group, which separated them from their customary and favorite study partners [22].

This study compared the learning effect of peer-assisted and faculty-led dissection, but it was limited in its ability to perform direct comparisons. First, we used different subjects at different times, so it might have had additional effects on the results. Although it was not available to compare the medical college admission tests or other parameters between two groups, the groups did not significantly differ with respect to basic characteristics; all entered the same medical school with similar levels of competence. Upper-limb anatomy education was investigated as the area of this research, which was presented by the same teacher in the unchanged curriculum from 2008 to 2012 and its outcomes were available for the evaluation in the same way between different time periods. Second, the assessment as an outcome measure was restricted to knowledge-based examinations. This study did not assess student performance such as individual dissection skills and peer group interactions. Another limitation was that student tutors had different levels of competence and were not prepared for peer tutoring through extra-education. As Cloward suggested, tutors do not have to be highly trained or excellent students [25]. Since student tutors are typically more cognitively congruent than a faculty, they tend to understand the problems their peers face and explain difficult concepts at their peers’ level of knowledge [9].

Despite these limitations, our results are still meaningful, as we used objective measures rather than merely students’ perceptions to compare the academic results of peer-assisted and faculty-led dissection. The learning outcome of peer-assisted dissection compared favorably with that of faculty-led dissection. Our study suggests several far-reaching benefits of peer-assisted learning. First, it can encourage students to participate more actively in the dissection process. Second, it is one of the effective learning methods to promote students’ academic achievement. Last, it should be an attractive alternative for the institution to alleviate the lack of faculty compared to the number of students as well.

## Conclusions

The students in peer-assisted learning perceived themselves as having a better understanding of course content and achieved better academic results. Students who serve as tutors are better able to acquire knowledge by teaching and tutees are encouraged to actively participate rather than act as bystanders. Institutions should offer programs that help students understand the meaning of peer-assisted learning, to learn how to become effective tutors, and to provide constructive feedback to peers.

## Supporting Information

S1 Table Dataset. (SAV)

S1 Text Anatomy Dissection Course Questionnaire. (DOCX)
