# Supplementary material for: Peer-Assisted Learning in a Gross Anatomy Dissection Course
Source: PLoS One. 2015 Nov 13;10(11):e0142988. doi: 10.1371/journal.pone.0142988 (PMC4643929; doi:10.1371/journal.pone.0142988)
Supplement: S1 Text — (DOCX) [file pone.0142988.s002.docx]

**Anatomy Dissection Course Questionnaire**

This questionnaire has been designed to identify how you regard the learning experience of anatomy dissection course. Please answer every question honestly by placing a mark or describing your opinion in the blank. Thank you for your participation.

| A. Demographic information |
| --- |

Student Number: Name:

Gender: □ Male □ Female Birth Year:

| B. The following scales are about how well you understand the learning objectives of upper-limb dissection. Please read each scale carefully and circle the number that best describes how much you can. |
| --- |

|  | Not  at all | A little bit | Some  what | Quite  a bit | Very much |
| --- | --- | --- | --- | --- | --- |
| 1. Describe the fascial compartments delimiting the major muscle groups of the upper limbs. | 1 | 2 | 3 | 4 | 5 |
| 2. Describe the origin, course and distribution of the major arteries and their branches | 1 | 2 | 3 | 4 | 5 |
| 3. Describe the courses of the main veins of the upper limbs | 1 | 2 | 3 | 4 | 5 |

| 4. Describe the organization of brachial plexus, its origin in the neck and continuation to the axilla and upper limb | 1 | 2 | 3 | 4 | 5 |
| --- | --- | --- | --- | --- | --- |
| 5. Describe the origin, course and function of the axillary, radial, musculocutaneous, median and ulnar nerves in the arm, fore-arm, wrist and hand | 1 | 2 | 3 | 4 | 5 |

|  | Not  at all | A little bit | Some  what | Quite  a bit | Very much |
| --- | --- | --- | --- | --- | --- |
| 6. Describe the boundaries of the axilla | 1 | 2 | 3 | 4 | 5 |
| 7. Describe the movements of the pectoral girdle | 1 | 2 | 3 | 4 | 5 |
| 8. Describe the factors that contribute to the stability of the shoulder joint | 1 | 2 | 3 | 4 | 5 |
| 9. Describe the anatomy of the elbow joint | 1 | 2 | 3 | 4 | 5 |
| 10. Describe the anatomy of the wrist | 1 | 2 | 3 | 4 | 5 |
| 11. Name and demonstrate the movements of the fingers and thumb | 1 | 2 | 3 | 4 | 5 |
| 12. Describe the position and function of the retinacula of the wrist and the tendon sheaths of the wrist and hand | 1 | 2 | 3 | 4 | 5 |

| C. During the dissection course, you assisted peers’ learning as a tutor or received peer tutor’s help as a tutee. Please describe your frank opinions about the interaction between a tutor and tutees. |
| --- |

1. **As a tutor**, please describe your opinions about the pros and cons of peer-assisted learning in the dissection course.

| **pros** | **cons** |
| --- | --- |
|  |  |

2. **As a tutee**, please describe your opinions about the pros and cons of peer-assisted learning in the dissection course.

| **pros** | **cons** |
| --- | --- |
|  |  |
